# Cognitive mapping to decode farmers' mindsets in agricultural decision-making: a systematic review

**DOI:** 10.3389/fcogn.2026.1686530

**Published:** 2026-02-16

**Authors:** Arunachalam Panchatcharam SriVidhya, Paul J. Mansingh

**Affiliations:** Department of Agricultural Extension and Economics, VIT School of Agricultural Innovations and Advanced Learning (VAIAL), Vellore Institute of Technology, Vellore, Tamil Nadu, India

**Keywords:** cognitive mapping, agriculture, farmers, behavior, decision-making, TCCM

## Abstract

**Background:**

Farmers decision-making processes are critical to the implementation of technologies and climate-resilient sustainable practices. These decisions are subject to complex thinking processes, which include risk perception and belief systems. This systematic review explains the decision-making process of farmers, through cognitive mapping. Moreover, it examines mental models, perception, belief system and cause-effect relationship of farmers with particular attention given to their behavior and practices.

**Methods:**

The research adopted the Preferred Reporting Items for Systematic Reviews and Meta-Analysis (PRISMA) framework. It utilized Scopus and Web of Science databases to retrieve 80 articles. The main aim was to identify research trends and gaps through bibliometric analysis and the TCCM framework by focusing on major theories, contexts, characteristics, and methodologies of agricultural cognitive-mapping research.

**Results:**

The major trends identified in this study are risk perception in agricultural economics, climatic change, technology adoption, conservative agricultural and sustainability. The major stakeholders considered included farmers (cattle, pig, rice, and date farmers), extension agents, policymakers, NGOs, rural households, agro-industries, technology providers, and the holders of indigenous knowledge. There are still major gaps in understanding the psychological and cognitive processes underlying the decisions of farmers: longitudinal studies are limited, the role of gender is not studied thoroughly, particularly in the conditions of climate-change effects and policy shifts.

**Discussion:**

Though, the mental model, perceptions, and belief systems have a significant impact on agricultural decision-making, there are still gaps in the comprehension of psychological and cognitive mechanisms involved since it is a persistent problem in the agricultural decision-making process. Future studies must incorporate the behavioral psychology, mixed-methods and cross-cultural research designs to develop integrated models that support agricultural sustainability and resilience.

## 1 Introduction

Food security is sustained by agriculture and remains as the backbone of livelihood for billions worldwide. Currently, decision-making in agriculture has become increasingly complex because of rapid environmental fluctuations, technological advancement, and demands of socio-economic conditions. Uncertainties in farming especially due to climate change have affected the perception of farmers regarding risks and their adaptation strategies.

Smallholder farmers contribute to nearly 80% of the world's food production; however, they are disproportionately vulnerable to climate change and market instability (FAO, 2024). Thus, it would be crucial to find out the mindsets of farmers and thereby to develop policies that support the principles of sustainable agriculture. In this context, cognitive mapping, a popular tool in behavioral and social sciences provides a systematic measure toward understanding the thought process, beliefs, and behavioral making patterns of the farmers.

Cognitive mapping was defined by Edward Tolman in his article titled “Cognitive Maps in rats and men” to understand the mechanism by which rats navigate mazes (Tolman, 1948). His experiments proved the aspect of latent learning in which rats would absorb the layout of the maze in passive manner and without baiting and utilize the acquired knowledge aptly when an incentive of food is brought along. This idea was later transformed into a kind of mental model of real spaces and helped in navigation and choice. This psychological development of an insight into spatial navigation developed to deal with diverse applications in agriculture. Cognitive mapping is increasingly recognized as an effective tool for comprehending complex agricultural decision-making processes (Elsawah et al., 2015; Markinos et al., 2007). It is also helpful in improving farm management, extension services, policy-making, and promoting sustainable practices by understanding mental models (Van Winsen et al., 2013). By systematically analyzing the mental models of farmers, researchers can identify the key drivers of agricultural behavior and design effective support systems.

While the previous published reviews on cognitive mapping concentrated on conservation agriculture (Cicek et al., 2023), mental farming models (Eckert and Bell, 2005), and systems analysis tools (Ditzler et al., 2018), they are methodologically fragmented, and domain-specific (Table 1). The significance of the current systematic literature review is the attempt to generalize the current knowledge on cognitive mapping studies in agricultural decision-making and define gaps to be addressed in the future research. It is important to note that the literature has not explored how cognitive mapping can be integrated into farmer-focused decision systems in different geographies and farming systems. This critical gap is filled by reviewing existing literature of cognitive mapping and its use in agricultural decision-making processes, important themes, trends in methodology, and socio-environmental factors that drive cognitive perceptions of farmers. This focus on cognitive mapping is important as it offers an explanation of the influence of the mental image of the environments and practices by the farmers on the way decisions are made. The review provides theoretical understanding through the conceptualization of cognitive mapping that connects behavioral, socio-ecological, and systems perspective of agricultural decision making. It also gives practical understanding by demonstrating how the cognitive mapping tools have been used to design participatory decision support systems, customize policy interventions to local cognitive contexts. Such applications will play a critical role in helping farmers to become more resilient to rising socio-environmental uncertainty. The results will guide policy-makers, agribusiness practitioners, and providers of extension services to be able to encourage sustainable agricultural practices amid an increasing surreal socio-environmental uncertainty. With the scope mentioned above, the following research questions were explored in the present study,

(1) What are the major trends, influential authors, and thematic clusters in cognitive mapping research in agriculture?
(2) What are the key theories and methodologies used to study cognitive mapping in farmer decision-making?
(3) What social, economic, and environmental factors influence farmers' cognitive perceptions and agricultural choices?

**Table 1:** Summary of existing reviews on cognitive mapping in agriculture.

**Title**	**Contribution**	**Approach/ Methodology**	**Reference**
A critical assessment of conservation agriculture among smallholders in the Mediterranean region: adoption pathways inspired by agroecological principles	This review assesses the barriers to implementing Conservation Agriculture (CA) among farmers and stakeholders, addressing socio-cultural, economic, and biophysical challenges.	System analysis	Cicek et al., 2023
Invisible Force: Farmers' Mental Models and How They Influence Learning and Actions	This review explores the nature of mental farming models among small farm operators and their role in farming.	Qualitative study	Eckert and Bell, 2005
Affordances of agricultural systems analysis tools: A review and framework to enhance tool design and implementation	This review explores the implementation of systems analysis (SA) tools in agricultural settings, focusing on their use in participatory problem-solving processes using Fuzzy Cognitive Mapping (FCM), bio-economic whole-farm models (BEFM), and role play and serious games (RPSG)	System Analysis	Ditzler et al., 2018

**Source**: Author's compilation (2024).

## 2 Methodology

**Figure 1:**
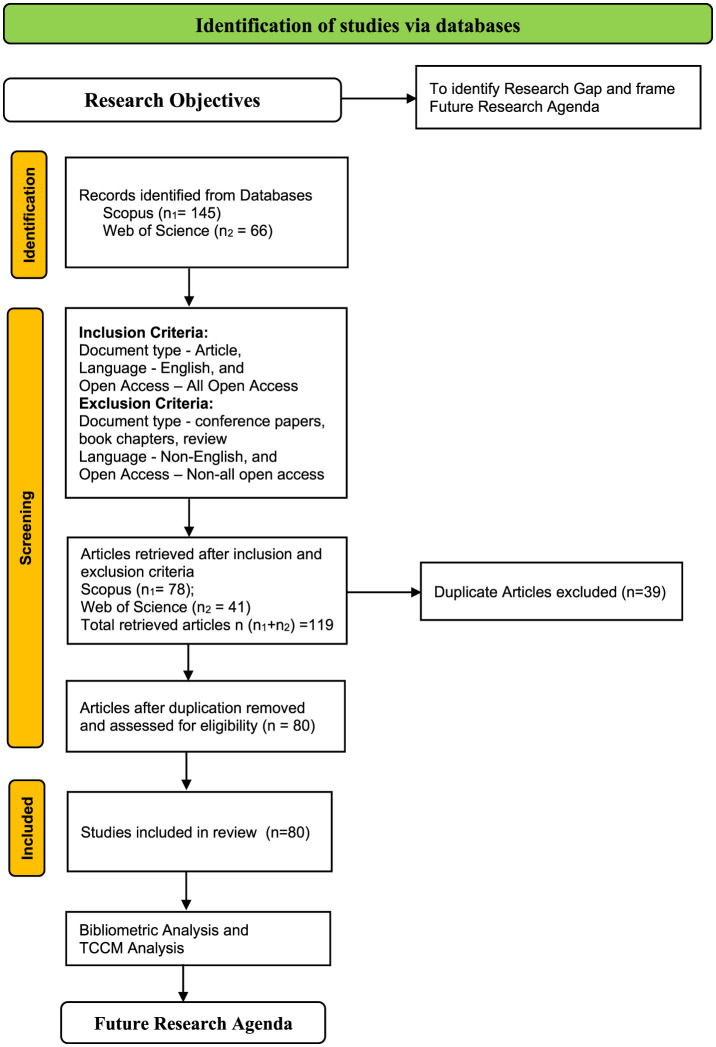
PRISMA framework. **Source**: Author's compilation (2024).

This review utilizes the Systematic Literature Review (SLR) approach to systematically identify, assess, and synthesize the existing literature on cognitive mapping in agriculture. The SLR adheres to the PRISMA (Preferred Reporting Items for Systematic Reviews and Meta-Analyses) guidelines (Figure 1). Relevant literature was searched on May 29, 2024, among two major online databases: Scopus and Web of Science. The databases were searched using the search string (“Cognitive Mapping” OR “Mental Model”) AND (“Farmers” OR “Cultivators”). A total of 211 documents (145 from Scopus and 66 from Web of Science) were retrieved from both databases. The search was limited to the predefined inclusion and exclusion criteria to ensure the quality of the selected articles. The inclusion criteria included studies that were focused on cognitive mapping in agriculture, document type (article), language (English), and open access (all open access). Studies that were irrelevant to cognitive mapping, conference papers, book chapters, review articles, errata, non-English articles were excluded. These inclusion and exclusion criteria minimized the results to 119 documents (78 from Scopus and 41 from Web of Science). The removal of the duplicated documents was done using R Software that removed 39 documents. Due to elimination of the duplicate records, 80 studies remained and were critically analyzed in regard to quality and relevancy. The screening and selection process was done with utmost care that dwelled on cognitive mapping of agriculture to remove irrelevant studies, possible bias and consistency of the inclusion and exclusion criteria. The final screening had 80 published studies that were published between 2009 and 2024. Furthermore, bibliometric and TCCM framework analyses were conducted to observe research trends and themes and to identify critical gaps for future research in agricultural systems. R Studio and VOS viewer software were used for the bibliometric analysis because of their efficacy in advanced data visualization.

## 3 Results and discussion

### 3.1 Bibliometric analysis

**Figure 2:**
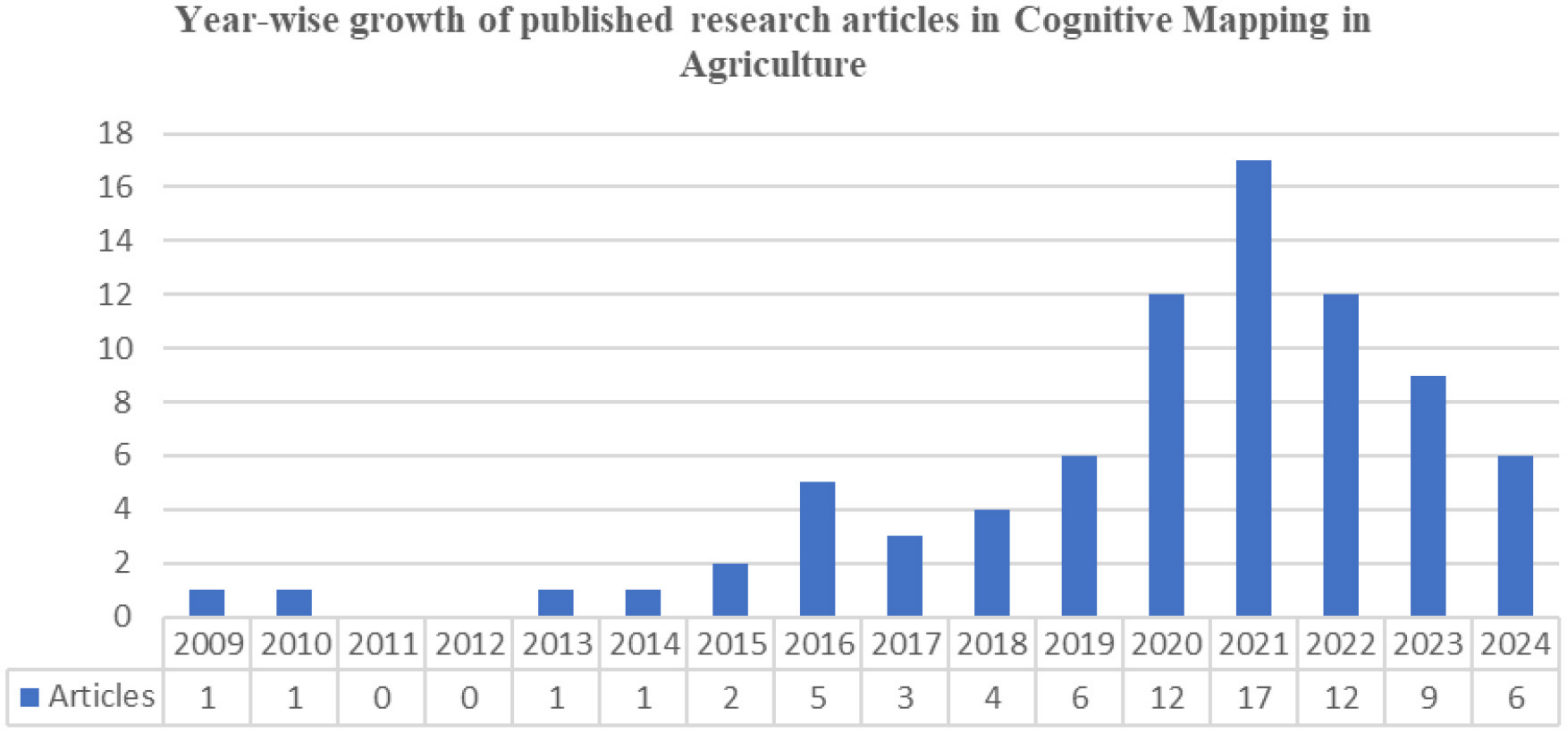
Year-wise growth of research on cognitive mapping in agriculture. **Source**: Author's compilation (2024).

Figure 2 shows the year-wise growth of research on cognitive mapping in agriculture. It was observed that between 2009 and 2015, the volume of published articles remained relatively low, indicating limited research activity and awareness about cognitive mapping. Nevertheless, the significant increase since 2015 and 2017 suggests the emergence of the scholarly awareness of systems thinking, participatory modeling, and the necessity to elicit the mental models of farmers in the studies of sustainable agriculture. It was observed that cognitive mapping was used as a tool to elicit the perceptions of farmers and to support improved decision-making. A peak of research was observed in 2021, which indicates a growing scholarly recognition and interest in decision-support tools and resilient studies following global disruptions such as the COVID-19 pandemic, which intensified the interest in understanding farmer's adaptive behaviors. Following 2021, a decline in publication numbers was observed during the post-COVID era, the continued activity from 2022 to 2024 indicates that cognitive mapping has evolved into an significant area of research interest in agricultural decision research within agricultural systems studies.

**Figure 3:**
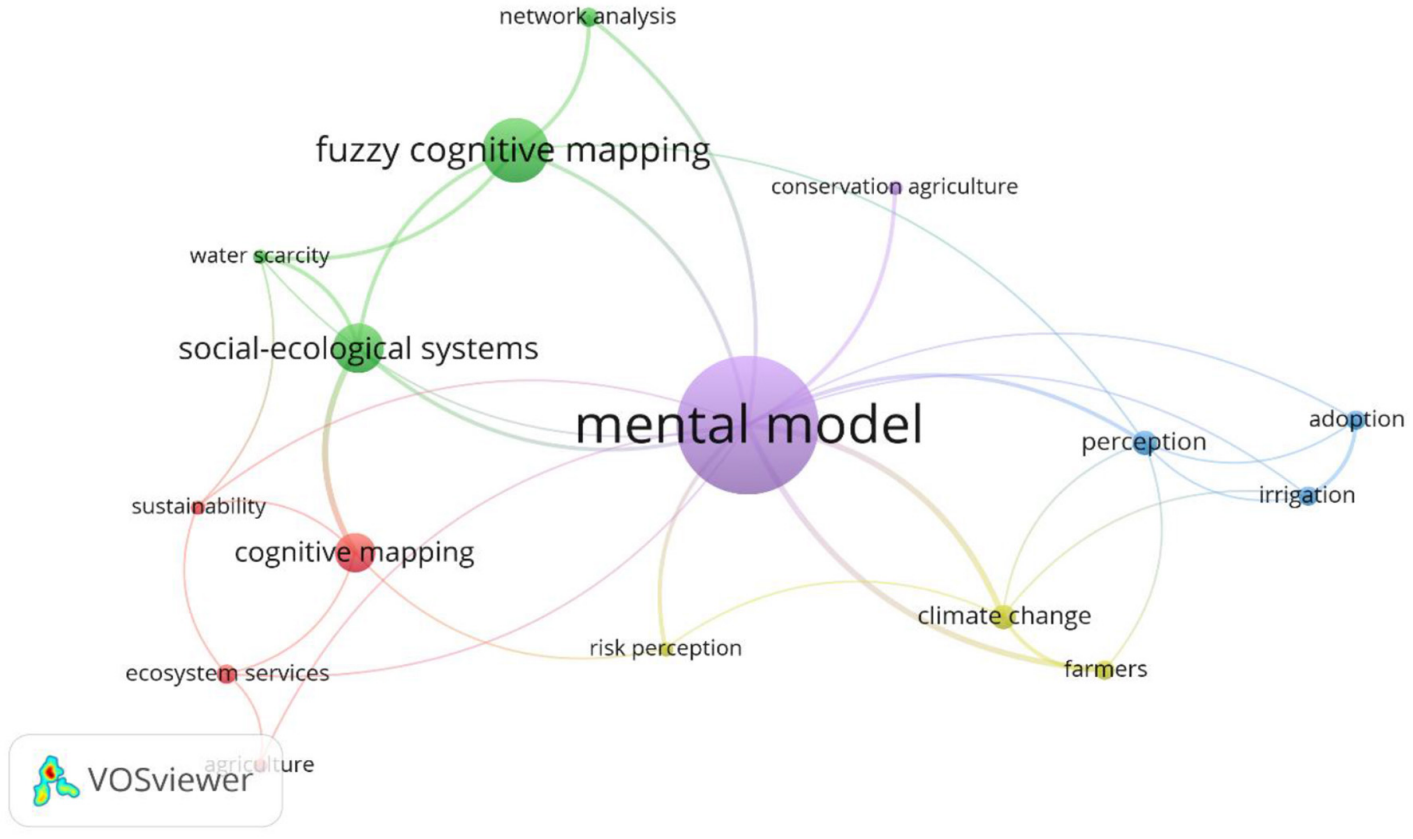
Keyword co-occurrence analysis of authors' keywords. **Source**: Author's compilation from Scopus and WOS bibliometric data (2024).

Figure 3 shows the Keyword co-occurrence analysis, which is an effective tool of revealing the new themes of research (Kevork and Vrechopoulos, 2009). The VOSviewer software was used to perform keyword co-occurrence analysis as it is effective in developing and visualizing bibliometric networks. The most recurring themes are mental model, cognitive mapping, social-ecological systems, fuzzy cognitive mapping (FCM), adoption, perception, and risk perception. These themes strongly focus on farmers' and stakeholders' cognitive frameworks within decision-making contexts. FCM is a cognitive map that visualizes mental models and causal connections by incorporating fuzzy logic. Studies focused on “Social-ecological systems” highlight the growing recognition of the complex interactions between human behavior and ecological processes in agricultural contexts. Themes like perception indicate individual and collective evaluations in shaping agricultural practices. These findings demonstrate the advancing cognitive mapping research in developing decision support models and adaptive governance in agricultural policy-making.

**Figure 4:**
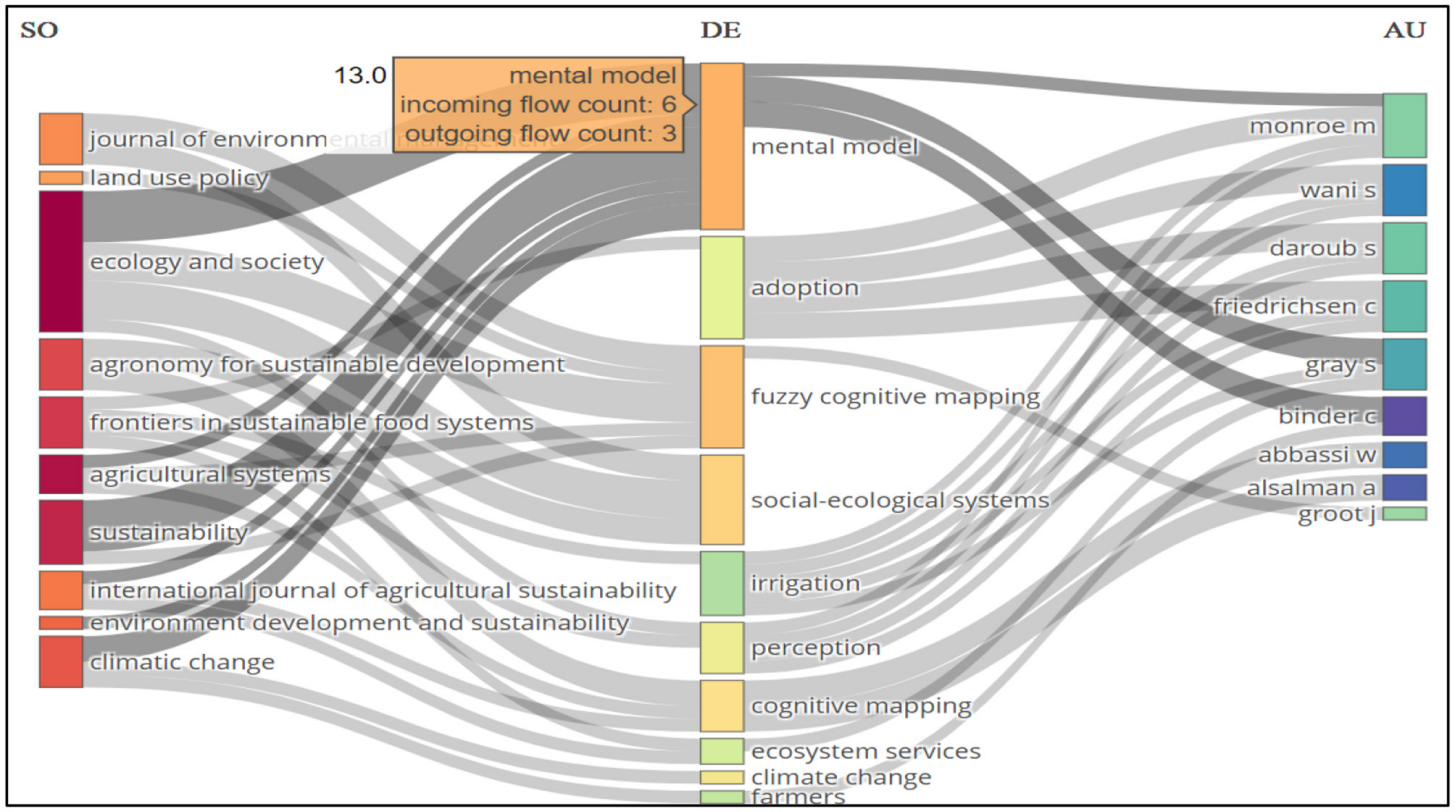
Three field plot (Sankey diagram) showing relationships among the major source journals (SO), descriptors/keywords themes (DE), and Authors (AU) in cognitive mapping research. **Source**: Author's compilation from Scopus and WOS bibliometric data (2024).

Figure 4 depicts the three-field plot that is used to visualize the interconnectedness and relationships between authors (right), Keywords/descriptors (Center), and Journals (Left). It is commonly referred to as Sankey diagram that provides valuable insights into the structure and dynamics of a research domain. The height of the rectangle displays the correlation between specific journals, keywords, and authors. The results show that the key prominent journals include Ecology and Society, Sustainability, Agricultural Systems, and Agronomy for Sustainable Management, which have frequently published research in cognitive mapping. Keywords such as the mental model, social-ecological systems, fuzzy cognitive mapping, adoption and perception represent the conceptual focus on the understanding of the cognitive frameworks of farmers in their agricultural decision-making. These keywords point to a growing emphasis on cognitive issues in an agricultural environment. The keyword, ‘adoption' signifies a behavioral outcome, which relates to adopting sustainable practices. It also symbolizes the increased focus on participatory and systems-thinking strategies. The authors most closely related to research of the mental models are Monroe M., Wani S., and Daroub S., and Friedricksen C. and Gray S., are involved in the research on fuzzy cognitive mapping. Their research relates cognitive mapping and sustainability, risk perception, and adoption. This synthesis attracts attention to the possible collaboration and dynamic goals of research that could be leveraged to facilitate a more effective perception of cognitive processes which facilitate sustainable agricultural decision making.

**Figure 5:**
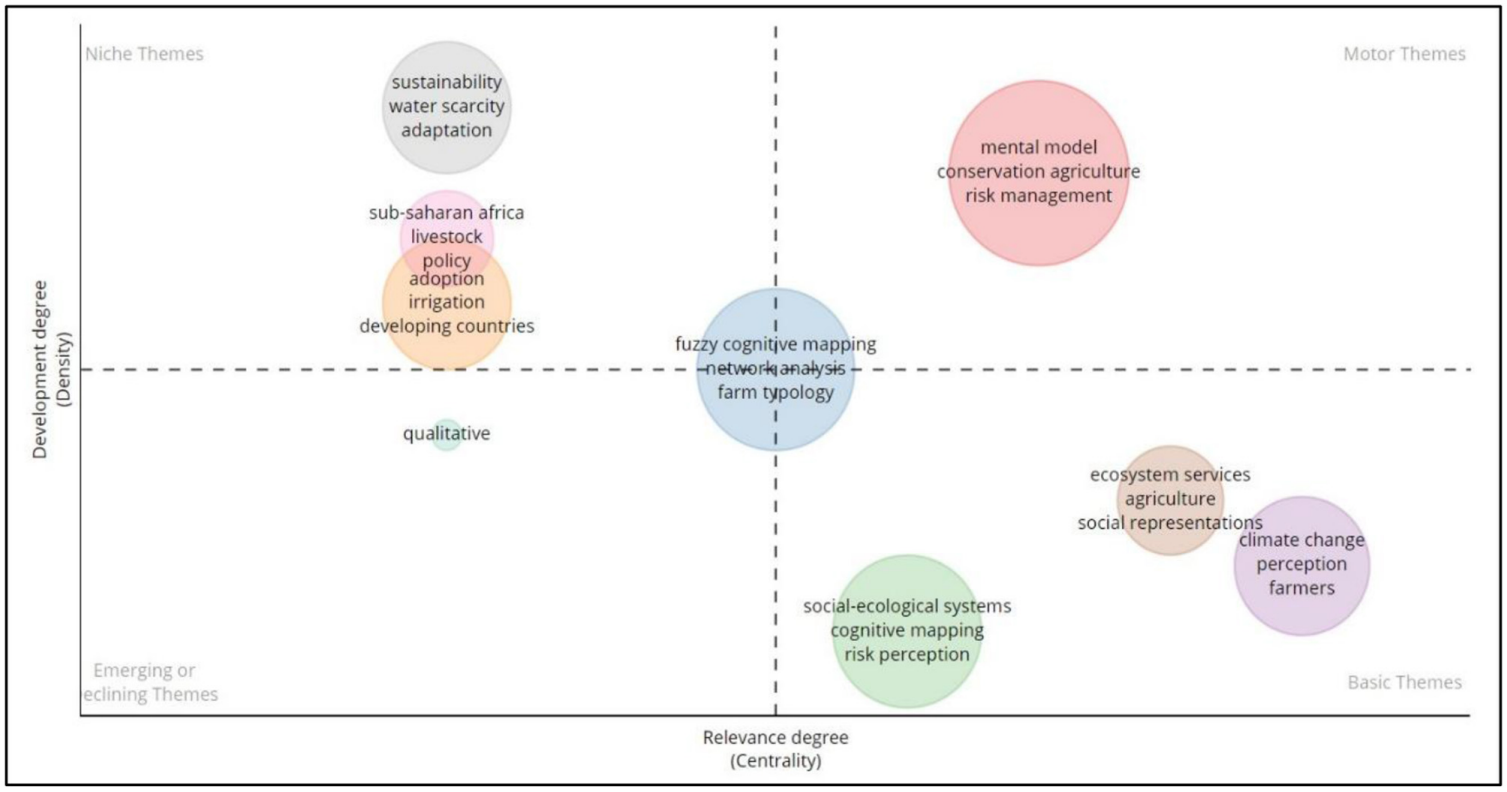
Major research themes in cognitive mapping research in agriculture. **Source**: Author's compilation from Scopus and WOS bibliometric data (2024).

Figure 5 illustrates a thematic map showing the major research themes in cognitive mapping research in agriculture. The size of the bubble indicates the number of documents per research themes according to their development (density) and relevance (centrality). It is evident that significant research is done in the motor themes including risk management, conservation agriculture, and mental modeling. They are highly relevant and drive research and policy discussions. Basic themes represent foundational constructs in the literature that are highly relevant but not as extensively developed, such as ecosystem services, agriculture, social representations, climate change, perception, and farmers. These basic themes require further research to enhance their development. Niche themes, such as sustainability, water scarcity, adaptation, livestock, policy, adoption, and irrigation, offer specialized insights and lower relevance in the broader research landscape. Emerging or declining themes, such as qualitative studies, have lower development and relevance, suggesting that they are gaining or losing significance. Fuzzy cognitive mapping, farm typology, and network analysis appear to be central and moderately developed, and therefore have potential for various research applications.

### 3.2 TCCM Framework

**Figure 6:**
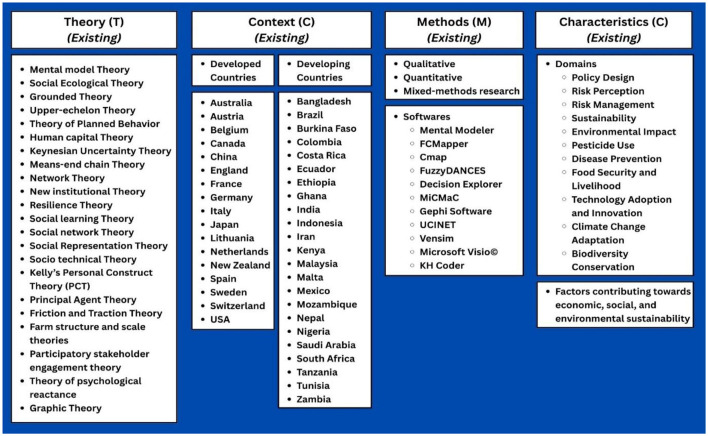
Application of Theory (T), Context (C), Characteristics (C), and Methods (M) framework. **Source**: Author's compilation from Scopus and WOS data (2024), developed for this study with insights from (Paul and Rosado-Serrano 2019), and Marc Lim et al. (2021).

This study utilized the TCCM framework developed by Paul and Rosado-Serrano (2019) to organize and synthesize the literature on cognitive mapping in agricultural decision-making (Figure 6). TCCM refers to theory (T), context (C), characteristics (C), and methodology (M). The results of the TCCM will address the critical gaps in the existing literature and offers guidance for future research.

#### 3.2.1 Theory (T)

**Figure 7:**
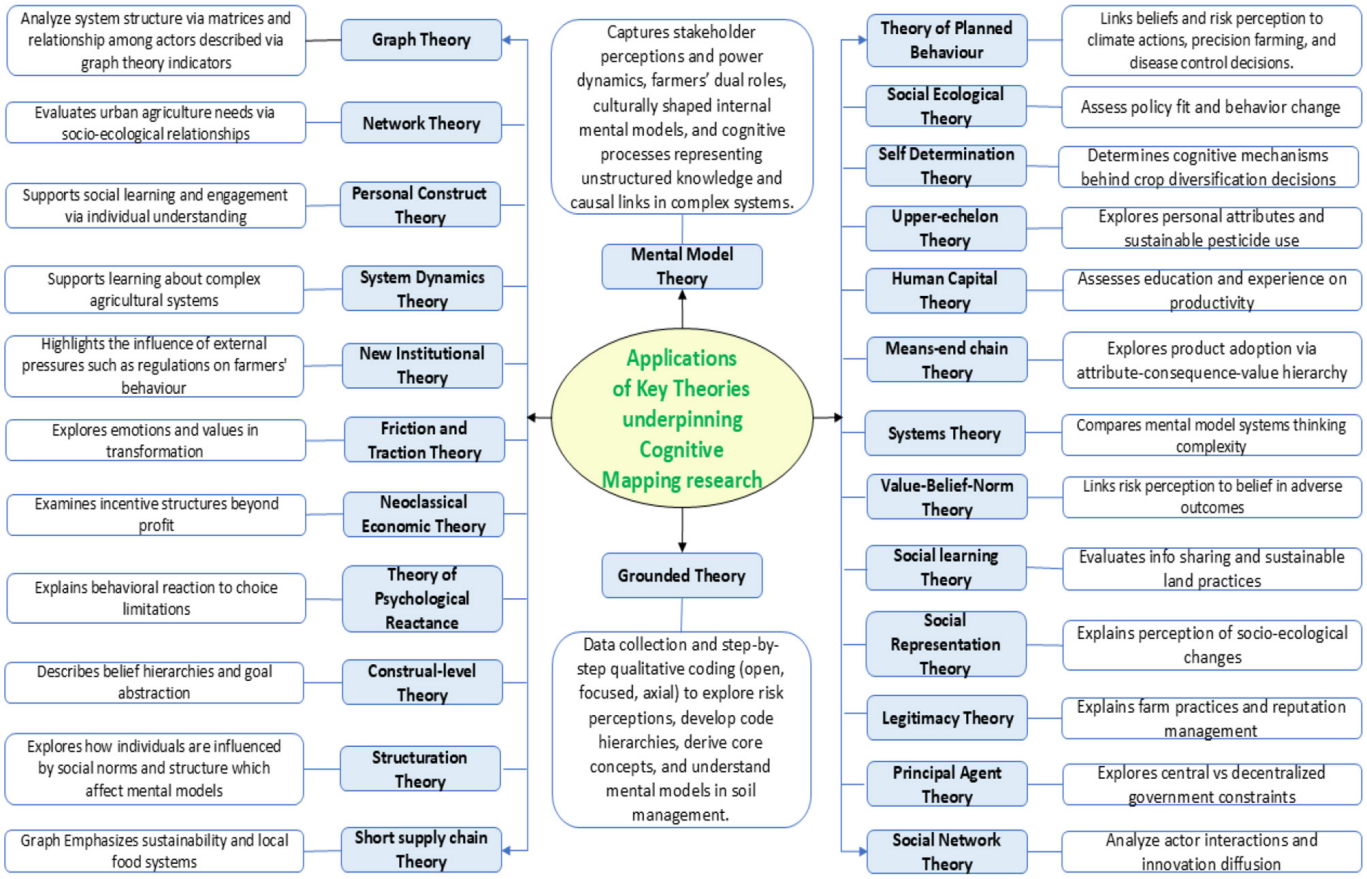
Application of key theories in cognitive mapping research in the agricultural sector. **Source**: Author's compilation (2024).

Figure 7 illustrates varying theories that substantiate the theoretical grounds in the field of cognitive mapping in agriculture. The theory that is most commonly used is the graph theory, which is crucial to investigate fuzzy cognitive maps and social cognitive maps. Graph theory enhances the interpretability because it reduces the complexity of the cognitive maps. It can also be used to compare FCMs with the Driver- Pressure-State-Impact-Response framework, to categorize variables that affect the socio-ecological system (Mehryar et al., 2017). It offers a structured perspective to examine the interrelations between cognitions through visualizing a cause and effect relationship between the system concepts (Satama and Iglesias, 2020; Edwards et al., 2023). The two indicators of the graph theory that is used to synthesize individual cognitive maps are relationship weight and actor centrality (Cornu et al., 2023). The most widespread approach that relies on graph theory is exploratory network analysis, where cognitive maps are transformed into adjacency matrices, where nodes are located on both axes (Micha et al., 2020). Mirroring of individual and group perceptions of social realities shall be connected with linking them as a network of nodes and edges (Müller et al., 2019). Graph-theory measures including the level of hierarchy, the value of centrality, outdegree, and indegree are common measures to analyze questionnaire-based network (Targetti et al., 2021). This allows the opportunity to examine the structure of a system in respect to emergent behavior as opposed to discrete units (Alomia-Hinojosa et al., 2023). Mental Modeler software uses graph-theory to estimate the strength and occurrence of influence between variables (outdegree centrality) and influenced variables (indegree centrality) (Steger et al., 2021).

Another most commonly theory used is grounded theory that helps in investigating the perceptions of the stakeholders without any presumptions (Van Winsen et al., 2013; Krithika et al., 2023). It enables the interpretation of qualitative data with the help of open and focused axial codings and extract basic core hierarchies in a systematic manner (Prager and Curfs, 2016; Kopainsky et al., 2017; Müller et al., 2019; Goswami et al., 2020; Cornu et al., 2023). Mental model theory on the other hand has contributed significantly to the analysis of the difference and similarities of the perceptions of farmers. For instance, when the perception of biogas systems is not same, it may become a barrier to further development, and therefore targeted support is suggested (Asai et al., 2019). The theory also provides an opportunity to achieve plural valuation of constructed wetlands by stakeholders. This allows gathering of various perceptions and help in navigating communication and power across gaps and overlaps among the groups of stakeholders (Friedrichsen et al., 2020, 2021). It also foster mutual knowledge of the mental models of agro-social-ecosystems by different stakeholders (Vuillot et al., 2016). The implementation of disease control among farmers was comprehended using the Theory of Planned Behavior (Suit-B et al., 2020). It assumes that the behavior intentions are defined by the beliefs, norms, and controls, thus connecting cognition with the behavior (Akimowicz et al., 2022). The theory offers an effective conceptual framework, which can be used to analyze the decision-making process of farmers in the management of irrigation operations (Monteleone et al., 2024). The theory of planned behavior has also been applied in adoption rates to get knowledge about the attitudes of farmers toward sustainable practices (Friedrichsen et al., 2018). Mental model elicitation was based on the social ecological system theory, using perception of a farmer to know the vulnerability of the social-ecological systems and environment barriers to adaptation (Wagner et al., 2020). This contributed to the achievement of the accuracy in capturing the adoption barriers in biochar implementation systems (Müller et al., 2019).

Several additional theories such as the human capital theory postulates that the investments made in education, training and experience may increase the productivity of an individual and lead to his or her success (Mohamed et al., 2024). This is applicable to farmers who invest in sustainable farming practices which will be more productive and profitable in the long run (Mohamed et al., 2024). New institutional theory suggests that stakeholders' behavior within a system is influenced by social norms, values, and regulations, beyond individual preferences and beliefs (Mohamed et al., 2024). Legitimacy theory suggests that farms must maintain legitimacy in the eyes of stakeholders to survive and thrive. Farmers maintain legitimacy with consumers, regulators, and other stakeholders, driving them to adopt pro-environmental behavior and environmentally sustainable practices (Mohamed et al., 2024). Means-End Chain (MEC) theory, drawn from economic psychology is applied to draw individual's perceptions of a product's features and characteristics (Okello et al., 2019). Network theory has revealed that networks function more efficiently when structured as platform ecosystems, replacing traditional market economies (Lankauskiene et al., 2022). It is also used to identify critical components and relationships in urban agriculture, particularly in evaluating the feasibility of a green label within vulnerable socio-ecological systems under COVID-19 restrictions (Arroyo-Lambaer et al., 2022). Theory of psychological reactance explains the strong concerns of conventional farmers about insect friendly farming practices (Busse et al., 2021). Self-determination theory was utilized to understand the degree of internal desires vs. external pressures (Cornu et al., 2023). Value-belief-norm theory holds that risk perceptions are tied to belief in the likelihood of adverse consequences. Construal-level theory suggests that hierarchical belief systems contain abstract, superordinate goals linked to subordinate beliefs about actions needed to achieve them (Hoffman et al., 2014). Kelly's personal construct theory has illustrated coherent storylines of adaptation delta management responses to community livelihood adaptation under uncertainty (Kulsum et al., 2020). Social representation theory, a socio-psychological theory has been applied to explain how different social groups develop varied understandings of issues based on their values, ideas, knowledge, beliefs, and practices (Vuillot et al., 2020). Social learning theory is applied through participatory monitoring and evaluation to enable learning, adoption, and out-scaling of regenerative agriculture (Soto et al., 2021). Social network theory uses model-based approaches to describe actor proximity and innovation diffusion dynamics, taking a non-linear perspective on interactions within relationship groups (Cornu et al., 2023). Systems theory was used to compare degrees of systems thinking, measuring complexity, non-linearity, cyclic Interdependence, and feedback representation across clusters of mental models (Lalani et al., 2021). The system structure (FCM) was compared and matched with the structural pattern and dynamic theory of generic system archetypes (Edwards et al., 2023). Giddens' structuration theory states that individuals are influenced by social structure-social norms, values, and rules in two ways: directly within their human capital and culturally specific traditions, and indirectly through their perception of livelihood and mental models (Binder and Schöll, 2010). It explains the structure of incentives beyond profit-making goals, as usually assumed by neoclassical economic theory (Okello et al., 2019). The incorporation of service-dominant logic in food supply chain management theory suggests a production strategy that allows consumers to purchase products customized to their requirements (Lankauskiene et al., 2022). Friction and Traction theory is applied to explore change and persistence in transitions to climate-smart regenerative agriculture (Gosnell et al., 2019). Managers of natural resource management projects often find it difficult to anticipate outcomes despite having an explicit change theory guiding them (Goswami et al., 2024).

The theoretical gap lies in the fact that these theories are never fused to form integrated cognitive maps that incorporate an individual's mental construction along with more generalist system interactions, although theories do exist. Most cognitive mapping research employs theories without integration, interdisciplinarity, or cross-firm perspectives. Future studies should also utilize other cognitive theories such as the (a) cognitive dissonance theory (Festinger, 1957) that explains how conflicting beliefs or experiences reshape cognitive maps and decisions, (b) prospect theory (Kahneman and Tversky, 1988) that is useful to model risk perception and decision biases within stakeholder cognitive structures, (c) resilience theory (Garmezy, 1991) that examines how adaptive thinking and mental models contribute to system resilience, (d) adaptive co-management theory (Holling, 1973) that connects stakeholder learning and cognitive alignment with governance adaptation, (e) risk perception theory (Slovic, 1987) that deepens understanding of how perceived risks are encoded in cognitive maps, and (f) diffusion of innovation theory (Rogers, 1962) that explains how cognitive structures influence adoption of innovations. Researchers can enhance inclusivity, policy relevance, and understanding of evolving cognitive models of agriculture and decision-making by engaging underrepresented stakeholders and using digital participatory platforms.

#### 3.2.2 Context (C)

The Context (C) component of the TCCM framework highlights the application and circumstances in which it is carried out, namely, the countries and areas of application (Marc Lim et al., 2021; Paul et al., 2017). Research on Cognitive mapping in agriculture has been conducted in many developing and developed countries (Figure 6). Most studies were carried out in countries such as India, the United States, and France, and their contexts are given in Table 2. A wide range of diverse studies has been conducted in India including COVID-19 impact (Goswami et al., 2020; Krithika et al., 2023), biochar systems (Müller et al., 2019), sustainable groundwater management (Sanga and Koli, 2023), wetland Maintenance (Friedrichsen et al., 2020; Sanga and Koli, 2023), Soil management (Friedrichsen et al., 2018), and zero-tillage potato cultivation (Goswami et al., 2024). Research on impact of COVID-19 shows how farmers resilience toward crisis to uphold food security. These research studies emphasize a further scope of research on impact of COVID-19 on farmers of western countries, gendered cognition in decision-making, cognitive links between crop choice and household nutrition, and understanding of e-commerce platforms. In addition, some studies were conducted by collecting data from multiple European countries. However, Least Developed Countries (LDC) and Small Island Developing States (SIDS) are largely unexplored. These regions face distinct environmental, socio-economic, and governance challenges which impact sustainable agricultural practices. Therefore, future research should focus on these underrepresented and region-specific studies to contextualize the extensive implications of cognitive mapping for local conditions.

**Table 2:** Country-wise context of the studies in cognitive mapping in agriculture.

**Country**	**Context**	**Reference**
India	Impact of COVID-19 on smallholder agricultural systems	Goswami et al., 2020
	Biochar System in South India	Müller et al., 2019
	Sustainable Groundwater Management	Sanga and Koli, 2023
	Wetland Maintenance	Sanga and Koli, 2023
	Soil Management for Food Security	Friedrichsen et al., 2018
	Food Security Resilient Strategies of Farm Households Post COVID-19	Krithika et al., 2023
	Zero-Tillage Potato Cultivation	Goswami et al., 2024
	Wetland Maintenance in semi-arid India	Friedrichsen et al., 2020
United States of America (USA)	Biological Pest Control	Bardenhagen et al., 2020
	Food Safety Risks	Parker et al., 2016
	Farmers' Perceptions of Climate Change Risk	Han et al., 2022
	Climate Change	Clements et al., 2021
	Sustainable Agriculture	Hoffman et al., 2014
France	Rural forest systems	Blanco et al., 2019
	Landscape and Biodiversity	Salliou and Barnaud, 2017
	Social Representations of the Landscape	Vuillot et al., 2020
	Land Management	Vuillot et al., 2016

**Source**: Author's compilation (2024).

#### 3.2.3 Characteristics (C)

**Figure 8:**
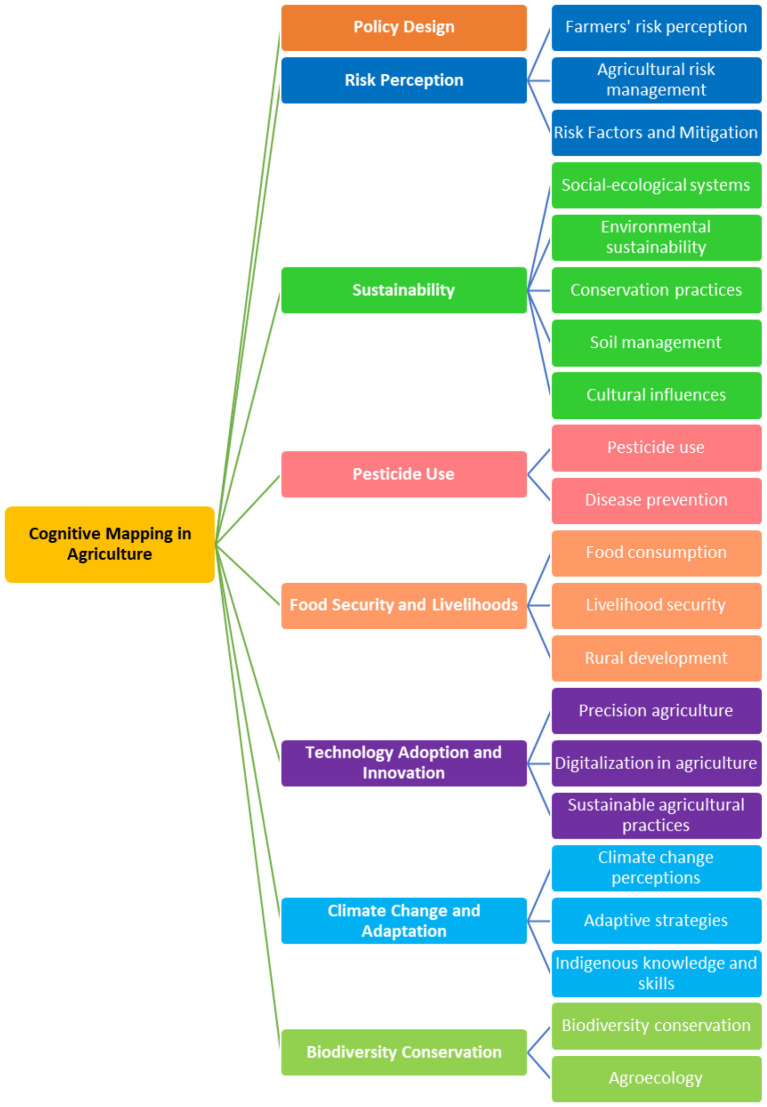
Major domains of application of cognitive mapping in agricultural decision making. **Source**: Author's compilation (2024).

Cognitive mapping and mental models provide insights into farmers' perceptions, decision-making processes, and adaptive responses to social, economic, environmental, and policy influences. The major stakeholders studied were farmers (cattle, pigs, rice, and dates), extension agents, policymakers, NGOs, rural households, agro-industries, technological solution providers, and custodians of indigenous knowledge. Figure 8 illustrates the diverse domains and subdomains of cognitive mapping in agricultural decision-making.

##### 3.2.3.1 Policy design

Agricultural policy design and implementation necessitate a nuanced understanding of the complex relationships between stakeholder dynamics and socio-ecological systems. The FCM integrates stakeholder positions, policy analysis simulations, and evaluations of alternative governance instruments for sustainable agriculture (Targetti et al., 2021). It revealed that the farmers place a higher value on bureaucracy and costs, whereas policymakers place a higher value on compliance and education (Christen et al., 2015). A good alignment of the beliefs of farmers with those of institutions (Akimowicz et al., 2022). The combination of FCM and the Driver-Pressure-State-Impact-Response (DPSIR) framework helps in the policy analysis of highly complex socio-ecological systems (Mehryar et al., 2017), whereas participatory system dynamics modeling improves policy design by refining the resilience of small-scale farmers (Kopainsky et al., 2017).

##### 3.2.3.2 Risk perception

Farmers tend to think of risks as dynamic and connected webs and not as separate events, severing risk assessments. High-intensity livestock production has created controversy among interested parties, and the varying views of farmers on its effect on people and animal wellbeing have been observed to be extremely high (Eijrond et al., 2023). Cognitive mapping helps diversify risks into a unified framework, making the judgment more informed and robust (Binder and Schöll, 2010). FCM, as elucidated by policymakers, farmers, and researchers, helps in communication and decision-making when perceptions are complex (Binder and Schöll, 2010; Vuillot et al., 2020). Dutta et al. (2023) proposed a new methodology called FCMCSO-ASC of Automated Soil Classification (ASC) based on combining FCM and Cat Swarm Optimization (CSO) with Local Diagonal Extrema Pattern (LDEP) to use feature extraction based on various types of soil and achieved 96.84 percent accuracy based on simulations (Dutta et al., 2023). The mental models of conservation agriculture farmers and conventional farmers significantly vary. The conservation agriculture farmers link good soil management with soil structure, fertility, and conservation, whereas conventional farmers focus on tillage and mineral fertilizers. Both groups value productivity and experience/knowledge highly, but conservation agriculture farmers link productivity to environmental responsibility and awareness (Topp et al., 2024). FCM was utilized to explore the control of late blight of potato in the Netherlands, and the results revealed risks identified included environmental damage by fungicides and social disputes between agrarians (Pacilly et al., 2016). The decision-making spaces in agricultural risk management through a mental model approach helps to reduce collective drought risk management (DRM) (Hanger-Kopp and Palka, 2022). Farmers were in a position of having difficulty striking a balance between the need to implement effective DRM measures, regulations, and economic pressure. This shows that integrated policy designs are needed for various factors affecting farmers' risk management decisions.

##### 3.2.3.3 Sustainability

The behavior of farmers is shaped by the interaction of several factors including physical, economic, cultural and institutional, that underlines the importance of various policies to enhance sustainability and ecological preservation (Allen et al., 2018). The decision-making process of farmers in the pest management, soil fertility, water use, and ecosystem conservation has been studied with the help of cognitive mapping and mental models (Arroyo-Lambaer et al., 2022). They can also be utilized to identify future changes in livelihood security and environmental sustainability (Diniz et al., 2015), to assess the behavior of farmers on water quality regulations (Wagner et al., 2020), agent-based models for water scarcity (Mehryar et al., 2019), and effects of policy (Diniz et al., 2015). High-tech devices, including optimization algorithms, which combine with FCM, facilitate efficient environmental decision-making, and justify the significance of local knowledge (Dutta et al., 2023). Farmers' management practices influence their perceptions. In Vermont, USA, in Lake Taupo and Lake Rotorua, New Zealand, water quality management affects the behavior of farmers in different ways (Salliou and Barnaud, 2017). Good management practices of soil are directly tied to the enrollment in local extension programs. It works together toward achieving larger economic viability and environmental stewardship objectives (Hoffman et al., 2014). Privatization of resource and a decline in soil fertility undoubtedly impact agricultural sustainability adversely (Dossouhoui et al., 2023). Research indicates that policies should integrate culture, beliefs, values, and conventions of farmers to ensure there are sustainable agricultural practices (Sanga and Koli, 2023).

**Figure 9:**
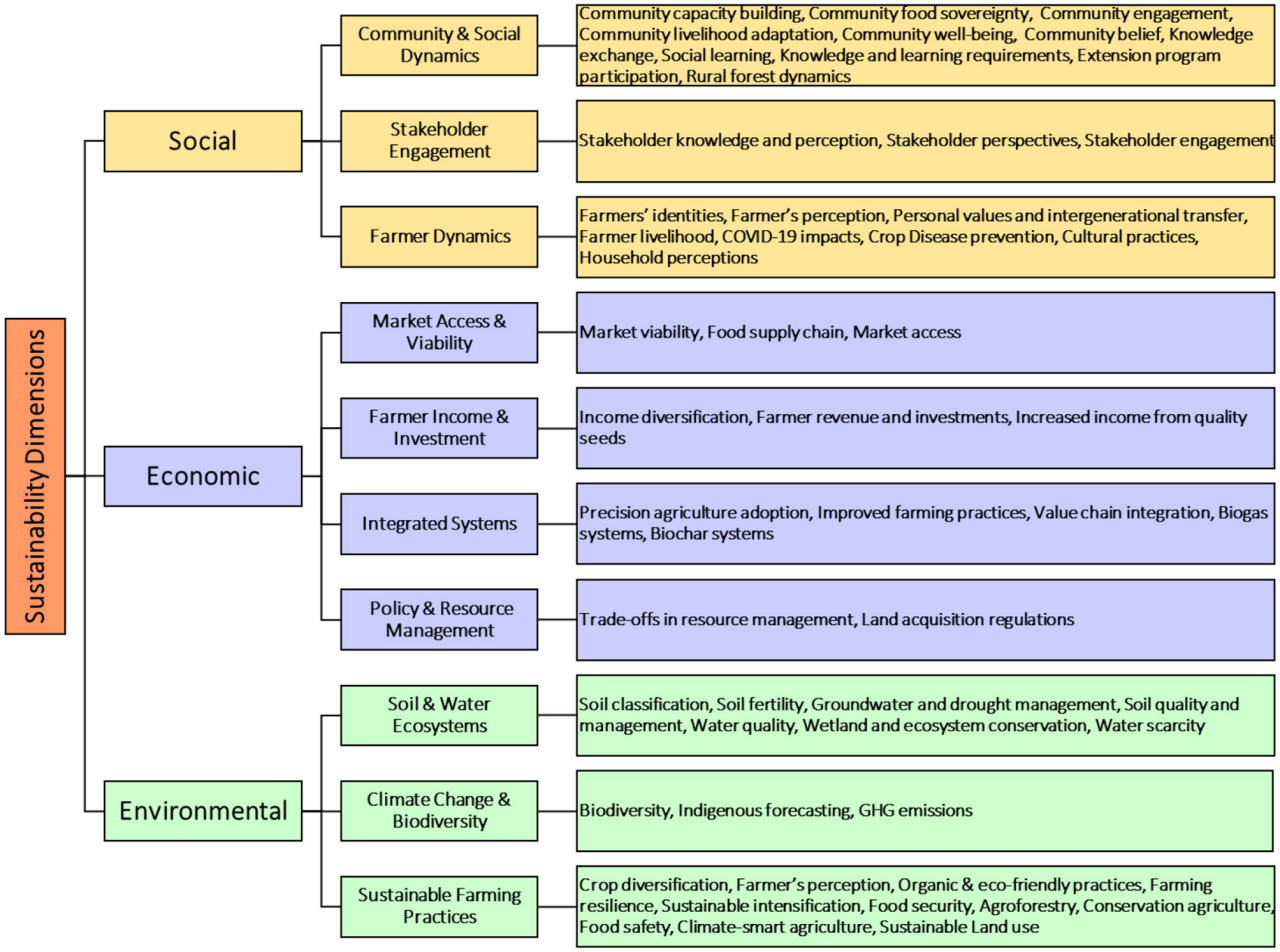
Key drivers of sustainability in agricultural decision making. **Source**: Author's compilation (2025).

Several studies have shown the significance of the important key drivers that contribute to the economic, social, and environmental aspects of sustainability in agro systems (Table 3 and Figure 9). Behavioral factors play a critical role in defining the modes of action in practices, i.e., in pesticide management (Ben Mohamed et al., 2024), disease prevention (Suit-B et al., 2020), and soil fertility (Yageta et al., 2022). Economic sustainability is connected with household disposable income, crop diversification, and food security (Diniz et al., 2015). Improved irrigation practices are considered as ways of ensuring environmental sustainability. Mental models of sustainability of farmers are hierarchically organized with concrete strategies on the periphery and abstract goals at the center. Such models are uniform across the regions, and those farmers incorporating more centralized concepts in their definitions have a higher chance of engaging in extension programs and adopting sustainable practices (Hoffman et al., 2014). In addition, studies have highlighted the integration of the various cognitive patterns of stakeholders in the process of developing particular forms of communication, training, and policy interventions (Suit-B et al., 2020; Villarreyna et al., 2020; Han et al., 2022; Eijrond et al., 2023). The fact that mental models are transforming food supply chains indicates that the resilience and sustainability of agriculture can be enhanced through the selection of innovative practices (Gosnell et al., 2019; Lankauskiene et al., 2022). There is also a need for more liberal sustainable model of agriculture, value of cooperation and an environmental policy that includes a cognitive sense to overcome the issues of the modern agri-food systems.

**Table 3:** Sustainability—dimensions, key themes, and key drivers in agricultural decision making.

**Sustainability dimension**	**Key themes**	**Key drivers of sustainability in agricultural decision making**
Social	Community and Social Dynamics	Community capacity building Goswami et al., (2024); Community food sovereignty (Arroyo-Lambaer et al., 2022); Community engagement (Kopainsky et al., 2017; Assogba et al., 2022; Lankauskiene et al., 2022); Community livelihood adaptation (Kulsum et al., 2020); Community wellbeing (Friedrichsen et al., 2020; Clements et al., 2021); Community beliefs (Adaawen, 2021); Knowledge exchange (Yageta et al., 2022); Social learning (Soto et al., 2021; Steger et al., 2021); Knowledge and learning requirements (Goller et al., 2021; Feser et al., 2021); Extension program participation (Hoffman et al., 2014); Rural forest dynamics (Blanco et al., 2019)
	Stakeholder Engagement	Stakeholder knowledge and perception (Pisanelli et al., 2023); Stakeholder perspectives (van Hulst et al., 2020; Eijrond et al., 2023); Stakeholder engagement (Douglas et al., 2016; Mikołajczak et al., 2022)
	Farmer Dynamics	Farmers' identities (Topp et al., 2024); Farmer's perception (Van Winsen et al., 2013; Busse et al., 2021; Alomia-Hinojosa et al., 2023); Personal values and intergenerational transfer (Okello et al., 2019; Akimowicz et al., 2022); Farmer livelihood (Schoell and Binder, 2009; Binder and Schöll, 2010; Lalani et al., 2021); COVID-19 impacts (Goswami et al., 2020; Krithika et al., 2023); Crop disease prevention (Pacilly et al., 2016; Suit-B et al., 2020); Cultural practices (Adaawen, 2021); Household perceptions (Yang and Zhen, 2020);
Economic	Market Access and Viability	Market viability (Mikołajczak et al., 2022); Food supply chain (Lankauskiene et al., 2022); Market access (Douglas et al., 2016)
	Farmer Income and Investment	Income diversification (Assogba et al., 2022); Farmer revenue and investments (Lalani et al., 2021; Akimowicz et al., 2022); Increased income from quality seeds (Okello et al., 2019)
	Integrated Systems	Precision agriculture adoption (Monteleone et al., 2024); Improved farming practices (MacLeod et al., 2022); Value chain integration (Wilson et al., 2021); Biogas systems (Asai et al., 2019); Biochar systems (Müller et al., 2019)
	Policy and Resource Management	Trade-offs in resource management (Alomia-Hinojosa et al., 2023); Land acquisition regulations (Hansson et al., 2021)
Environmental	Soil and Water Ecosystems	Soil classification (Dutta et al., 2023); Soil fertility (Dossouhoui et al., 2023); Groundwater and drought management (Masinde et al., 2018; Sanga and Koli, 2023); Soil quality and management (Friedrichsen et al., 2018; Lalani et al., 2021; Topp et al., 2024); Water quality (Wagner et al., 2020); Wetland and
		ecosystem conservation (Satama and Iglesias, 2020; Friedrichsen et al., 2021); Water scarcity (Mehryar et al., 2019)
	Climate Change and Biodiversity	Biodiversity (Busse et al., 2021; Mohamed et al., 2024); Indigenous forecasting (Nyadzi et al., 2021); GHG emissions (Asai et al., 2019; Satama and Iglesias, 2020)
	Sustainable Farming Practices	Crop diversification (Cornu et al., 2023); Farmer's perception (Daeli et al., 2021; Khumairoh et al., 2024); Organic & eco-friendly practices (Busse et al., 2021; Archibald et al., 2022); Farming resilience (Cordeiro et al., 2021); Sustainable intensification (Micha et al., 2020; Aravindakshan et al., 2021); Food security (Edwards et al., 2023); Agroforestry (Adaawen, 2021); Conservation agriculture (Lalani et al., 2021); Food safety (Parker et al., 2016); Climate-smart agriculture (Gosnell et al., 2019); Sustainable Land use (Christen et al., 2015; Vuillot et al., 2016)
Health	

**Source**: Author's compilation (2024).

##### 3.2.3.4 Pesticide use

Cognitive mapping has been an important agent in supporting the decision-making of a farmer on a farm level, by uncovering the factors that impact behavior of a farmer and making it possible to intervene. For instance, Ben Mohamed et al. (2024) and Suit-B et al. (2020), 2021) in their studies used cognitive mapping and discovered that the primary determinants of pesticides use are age, education, experience, farm size, moral norms, subjective norms, government regulations and personal attitude. These determinants have the indirect effect on the decision of farmers in the responsible use of pesticides. The combination of the individual mind maps to a common cognitive design can help design a context-sensitive interventions that fits the farmers mental models and thus increase the chances of adoption.

##### 3.2.3.5 Food security and livelihood

Cognitive mapping has been used in the context of food security and livelihood. Edwards et al. (2023) utilized FCM to analyze the rice agri-food system in Nigeria and identified systemic challenges and archetypes (e.g., failed remedies) affecting rice production. Yang and Zhen (2020) analyzed food consumption patterns in China using fuzzy cognitive mapping from the perspective of local rural areas. This study highlights the influence of livestock, income, and infrastructure on food consumption affects the differences in food security between pastoral and agro-pastoral zones. (Goswami et al., 2020) explored smallholder resilience during COVID-19 and Cyclone Amphan using fuzzy cognitive mapping and suggested adaptive measures and system dynamics for future resilience in India. Cordeiro et al. (2021) also analyzed smallholder resilience during the COVID-19 pandemic in Brazil and stated that financial and digital barriers affect food and livelihood security. Sustainable biochar systems and short food supply chains have been studied using FCM, emphasizing the importance of localized design and participatory planning to enhance food supply systems (Müller et al., 2019). Lankauskiene et al. (2022) considered the issue of rural sustainability through a mental model that addressed the food production and delivery system of post-industrial transitions to an agri-food system based on the Lithuanian platform of the Village to Your Home. This model concentrated on the role of consumers, network thinking, and service-dominant logic. Strategies such as adopting local perceptions and participatory policy making have been found to enhance rural resilience and livelihood security.

##### 3.2.3.6 Technology adoption and innovation

Monteleone et al. (2024) found that farmer behavior is an important factor in the irrigation operations management model using the Theory of Planned Behavior (TPB). The theory focuses on data-based decision-making and sharing in management practice. In addition to this behavioral perception, digitalization has revolutionized dairy farming in Germany, which has particularly changed farmers' knowledge and learning needs Goller et al., (2021). Different knowledge systems and perceptions of behavior can be harmonized using the Sustainable Intensification of Agriculture (SIA) to balance the interests of different stakeholders. Tackling SIA is an unavoidable process that necessitates the coordination of systems, collaboration of stakeholders, and policies that strengthen the sharing of experiences and farm resistance Micha et al., (2020). FCM provides insights into the complexity and dynamics of achieving SIA at the farm level. Aravindakshan et al. (2021) used FCM to explore the mental health models of farmers in coastal Bangladesh. These findings indicate that agricultural extension schemes, drainage, and microcredit are important for the adoption of SIA. Similarly, Wilson et al. (2021) deployed FCM to identify key integration points and challenges and prioritized local market networks and conducive policies to fortify food security. Cognitive mapping promotes the use of farm-level sustainability assessment tools. It provides intuitive decision aids and aligns with farmers‘ mental frameworks to improve their effectiveness MacLeod et al., (2022).

##### 3.2.3.7 Climate change and adaptation

Mental models from farming communities capture how farmers perceive climate risks and potential livelihood adaptations. This reveals how farmers make decisions in uncertain situations. In northeastern Ghana, local farmers interpret shifts in rainfall and drought based on indigenous knowledge and sociocultural beliefs Adaawen, (2021). Similarly, mental models have been used to quantitatively link indigenous ecological indicators to weather predictions. Farmers use mobile applications and rain gauges to record data, achieving accuracy comparable to that of the Ghana Meteorological Agency in daily forecasts but excelling at predicting seasonal rainfall cessation Nyadzi et al., (2021). FCM has also been recognized as an excellent tool for integrating indigenous knowledge of droughts with climate forecasts in Sub-Saharan Africa Masinde et al., (2018).

Rural communities face the challenge of maintaining a balanced relationship with their supernatural and physical surroundings Adaawen, (2021). South African commercial grain farmers use structured mental models to perceive and respond to climate-change risks. However, emotional and survival-oriented framings of weather and climate risks significantly hinder the adoption of climate-resilient practices, despite their potential benefits in mitigating both short- and long-term risks Findlater et al., (2018). Similarly, farmers' attitudes toward human exemptionalism and productivism influence their perceptions of climate risk Han et al., (2022). In India's Sundarbans, mental modeling and thematic analysis were used to assess the dual impact of COVID-19 and Cyclone Amphan on smallholder farmers. Key adaptations include utilizing family labor and early harvesting, with “Kharif” rice production and farm income as central factors Goswami et al., (2020). Robust social safety nets and stable income were studied in Tamil Nadu, India using fuzzy logic cognitive mapping. This study suggests interventions such as market accessibility and entrepreneurship development as solutions for stable income generation Krithika et al., (2023). In northern New England, mental modeling interviews have revealed differing views on the role of climate change in farming systems Douglas et al., (2016). Therefore, future studies can build on these findings by integrating both cognitive and socio-economic modeling approaches to capture a more holistic understanding of human-environment interactions.

##### 3.2.3.8 Biodiversity and conservation

The perception of farmers‘ about biodiversity plays an important role in conservation. A study in the northern Italian Alps found out that farmers' perception of biodiversity was characterized by relational mixed values, which are determined by their dual identities as producers and guardians of the landscape Moroder and Kernecker, (2022). German farmers‘ perceptions on the decline in insect biodiversity revealed mixed beliefs. Farmers were willing to adapt to insect-friendly practices but needed financial support. This underscores the role of shared social responsibility and policy reforms to effectively promote such measures Goswami et al., (2024). Agroecology has been indicated as a potential tactic to help meet the growing demand for economic, social, and environmental sustainability in agricultural systems van Hulst et al., (2020). Organic farming practices are key to the adoption of ecosystem services which positively enhance biodiversity. Farmers' perceptions are crucial for management decisions, that directly impact the sustainability and productivity of their farming systems Archibald et al., (2022). This also suggests for participatory dialogue to effectively promote improved landscape management and pest control strategies Salliou and Barnaud, (2017).

#### 3.2.4 Methods (M)

Cognitive mapping has been utilized in qualitative, quantitative, and mixed-methods studies. Qualitative methods include in-depth interviews, semi-structured interviews, cognitive mapping, ethnographic field studies, participant observations, and mental model analysis. These techniques are particularly helpful in understanding mental models, social factors, and stakeholder perceptions of complex issues, such as pesticide use Salliou and Barnaud, (2017); Busse et al., (2021); Ben Mohamed et al., (2024), rural forest change Daeli et al., (2021); Hansson et al., (2021), and food consumption patterns Arroyo-Lambaer et al., (2022). Quantitative methods are those which utilize structured surveys and statistical techniques to analyze data, although they are used less frequently. Mixed methods, which combine qualitative and quantitative methods, are employed extensively to understand farmers' perceptions and decision making across complex social, economic, and ecological systems Sandelowski, (2000). These research methodologies, utilized in various studies, enable researchers to investigate various aspects that influence effective agricultural decision-making processes (Table 4). This created a scope for integration between quantitative and qualitative modeling approaches. Future research shall adopt multi scale methods to enhance rigor and practical relevance.

**Table 4:** Sample size, region, and data collection tools utilized in cognitive mapping research.

**Reference**	**Sample size**	**Region**	**Data Collection Tool**
Van Winsen et al. (2013)	Nineteen farmers	Belgian provinces: Flemish Brabant and East Flanders.	Interview
Diniz et al. (2015)	Forty two small farmer households (Livestock-oriented (16 households); Diversified-oriented (13 households); Off-farm-oriented (13 households))	Southeastern Pará State, Eldorado do Carajás, Brazilian Amazon	Quantitative (factor and cluster analysis) and qualitative methods (content analysis, open-ended recorded interviews)
Ben Mohamed et al. (2024)	Eighty one date farmers	Qassim region of the Kingdom of Saudi Arabia	Semi-directive interviews
Suit-B et al. (2020)	Seven cattle farmers	Malaysia	Qualitative descriptive approach (semi-structured Interviews)
Mehryar et al. (2019)	Sixty farmers (20 in each category of small, medium and 186 large farmers) in	Rafsanjan, Iran	Qualitative (in-depth, open-ended interviews)
Schoell and Binder (2009)	Thirteen experts (5 females and 8 males) and 10 local farmers (all male)	Vereda la Hoya, Tunja, Departamento de Boyac'a (Colombia)	Qualitative (interviews)
Blanco et al. (2019)	Seventy forest owners and 210 forest users	*Vallées et Coteaux de Gascogne*, south-west France, Canton of Aurignac, Toulouse	Mixed methods (free-listings, semi-directive interviews, participative observation)
Yang and Zhen (2020)	Sixty one (21 families in West Ujimqin, 21 families in Zhenglan, and 25 families in Taibus)	Xilingol League, China	Face to face interviews
Suit-B et al. (2021)	Twelve pig farmers (mostly small-scale or medium-scale farms)	Negeri Sembilan, Melaka, and Johor (Malaysia)	Qualitative Approach (in-depth, semi-structured interviews)
Soto et al. (2021)	Twelve farmers (farmer association AlVelAl)	Steppe high plateau, semiarid southeast, Spain	Qualitative interviews
Aravindakshan et al. (2021)	Two hundred and forty farm households	Two districts—Patuakhali and Barguna, coastal Bangladesh	Survey
Moroder and Kernecker (2022)	Twenty two Farmers	Northern Italian Alps areas: Kastelruth, Predazzo, and Cortina d'Ampezzo	Structured telephonic interviews
Bardenhagen et al. (2020)	Thirty one Michigan blueberry and cherry farmers	Michigan State, USA	Questionnaires and cognitive maps
Cornu et al. (2023)	One hundred and twenty eight Crop Diversification Experiences	11 European partner countries and three climatic zones: (i) Atlantic, (ii) Mediterranean and Alpine South, and (iii) Continental, Pannonian, and Nemoral	Qualitative interviews and quantitative survey
Lalani et al. (2021)	Fifty Farmers	Metuge District, and Cabo Delgado, Mozambique	Focus group interview
Goswami et al. (2020)	Five selected stakeholders (2 farmers, 2 experts and 1 NGO staff)	Gosaba Block of South 24 Parganas district, West Bengal, Eastern India	Qualitative interview
Hanger-Kopp and Palka (2022)	Forty Austrian farmers	Austria	In-depth semi-structured interviews
Asai et al. (2019)	Twenty two stakeholders	Shihoro town, Northern Japanese	Case study
Mehryar et al. (2017)	Sixty individual farmers and 40 individual researchers and policy makers	Rafsanjan, Iran	Qualitative and quantitative analysis using DPSIR and FCM
Edwards et al. (2023)	Twenty three Stakeholders	Nigeria	Qualitative and quantitative analysis using fuzzy cognitive mapping and archetype analysis
Satama and Iglesias (2020) **s**	Twenty four participants	Ecuador, South America	Semi-quantitative approach (Focus group discussion, map analysis, and FCM construction)
Akimowicz et al. (2022)	Forty one Farmers	Two peri-urban areas in Canada and France,	Semi-structured interviews and mental modeling
Pacilly et al. (2016)	Twenty five Farmers (18 conventional and 7 organic farmers)	Netherlands	Semi-structured interviews
Dossouhoui et al. (2023)	One hundred and twenty six actors (farmers, herders, and extension agents)	Kandi, Pehunco, and Savalou (West Africa)	Semi-structured questionnaire and graphical support
Villarreyna et al. (2020)	Fifty eight Farmers	Nicaragua	Interviews with farmers, field observations in coffee plots
Masinde et al. (2018)	Two hundred respondents	Mbeere (Eastern Kenya), Nganyi, (Western Kenya), Muchenedze, (Mozambique), KwaZulu Natal (South Africa), and Taita-Taveta (Kenya)	Structured interviews, Case Studies and literature review
Dutta et al. (2023)	No respondents	Not a region/study specific study	FCMCSO-ASC technique incorporates local diagonal extrema pattern (LDEP) as a feature extractor for producing a collection of feature vectors.
Monteleone et al. (2024)	Twenty seven experts, 2 farms	Brazil	Interviews with experts, case studies on farms, modeling of irrigation operations management
Micha et al. (2020)	Forty eight people (farmers, advisors, social, agri-environmental scientists, and policy implementers)	European countries including Ireland, the UK, Belgium, Germany, Greece and France	Data were recorded during the workshop
Prager and Curfs (2016)	Sixteen (agricultural advisors, regional and local authorities, researchers)	Western Andevalo, Huelva province, South-western Spain	Semi-structured interviews
Salliou and Barnaud (2017)	Thirty (13 fruit growers, 3 livestock breeders, 3 cereal growers, 2 market gardeners, 6 crop advisors, and 3 landowners (mostly retired farmers).	Southwestern France	Semidirected and open-ended interviews
Kulsum et al. (2020)	Twenty rice farmers	Southwest coastal region of Bangladesh	Semi-structured interview, Focus Group Discussion (FGD), use of crop calendars,
Kopainsky et al. (2017)	Twenty small-scale farmers	Chibombo district, Central Province, North of Lusaka	In-depth interviews and discussions to identify the problem of interest. The workshop
Müller et al. (2019)	Fifty nine farmers	Chandagalu (Mandya district) and Manoor (Udupi district), Karnataka, South India	Qualitative interviews, focus groups, literature review, cognitive mapping
Archibald et al. (2022)	Hundred to three hundred shade trees and 9 farmers	Turrialba, Central Valley of Costa Rica.	Taxonomic and functional diversity measurement, semi-structured interviews, cognitive mapping
Assogba et al. (2022)	Two hundred and twenty eight households	Semi-arid zone, Burkina Faso	Statistical typology, participatory typology, household survey, focus group discussions, FCM
Lankauskiene et al. (2022)	Five (3 initiators and initial implementers and 2 network platform's administrative bodies)	Lithuania	In-depth interviews
Wilson et al. (2021)	Five hundred and sixty nine farming households, followed by a participatory workshop with 54 stakeholders	Southern Highlands (SH) of Tanzania; Iringa, Njombe and Ruvuma	Semi-structured interviews, and participatory workshop
Pisanelli et al. (2023)	Twenty four participants (practitioners, multipliers, researchers, suppliers and members of local administration)	Orvieto district, Central Italy	Stakeholder surveys, FCM mapping, scenario analysis
Arroyo-Lambaer et al. (2022)	Five agro-ecological chinampa producers	Xochimilco, Southern part of Mexico	Literature review, interviews, mental model elicitation, cognitive mapping
Sanga and Koli (2023)	Forty three farmers	Maharashtra, India	Qualitative open-ended interviews
Mohamed et al. (2024)	One hundred and twenty three date farmers	Qassim region, Kingdom of Saudi Arabia.	Questionnaire survey
Targetti et al. (2021)	Thirty stakeholders	Marchfeld, Eastern Austria	Focus groups, mind mapping, scenario co-development, individual interviews
Friedrichsen et al. (2021)	Semi-structured interviews (*n =* 39) and focus groups (*n =* 4)	Telangana and Karnataka, India	Qualitative data analysis, semi-structured interviews, focus groups
Cordeiro et al. (2021)	Two farmers and in a cooperative of family farmers	Rio de Janeiro State, Brazil	Case studies through semi-structured interviews and concept maps, data triangulation using literature review
Binder and Schöll (2010)	Four to five farmer interviews with a sample size of 10 or 20 persons	Colombia and Nicaragua	Interview and Structured Mental Model Approach
Vuillot et al. (2020)	One hundred and nineteen farmers	France (Camargue, Plaine et Val de Sèvre, Armorique, and Gascony Valleys and Hills)	Free-listing tasks, face-to-face interviews
Gosnell et al. (2019)	Twenty eight interviews	New South Wales (NSW), Australia	Semi-structured interviews, participant observation, and document analysis
Galli et al. (2022)	Twenty one farmers and 6 experts	Switzerland	Semi-structured interviews with experts and farmers, followed by an expert workshop
Parker et al. (2016)	Nineteen food safety experts and 32 produce growers	Indiana, Kentucky, Michigan, and Ohio	Mail survey, and semi-structured interviews
Vuillot et al. (2016)	Twenty six farmers	Coteaux de Gascogne territory, a hilly crop-livestock region in southern France	Face-to-face interviews with each farmer and Individual Mental Models (IMMs)
Daeli et al. (2021)	Two communities in three distinct landscape contexts (*n =* 24 participants per land- scape), and 72 interviews in total	Kapuas Hulu, Kalimantan,	Conceptual Content Cognitive Mapping approach, participant observation, individual interviews
Khumairoh et al. (2024)	One hundred and eleven farm households	Two districts of East Java Province, Indonesia: Malang and Lamongan.	In-depth Interviews, Farm Visits, Pilot Surveys, Focus group discussions
Goller et al. (2021)	Ten farmers, with 1 female among them, from nine different dairy farms	Germany: North Rhine-Westfalia and the south of Lower-Saxonia	Exploratory interviews using a semi-structured interview
Eijrond et al. (2023)	One thousand four hundred and twelve [livestock dense municipalities (*n =* 808), farmers (*n =* 237) and other stakeholders (*n =* 367)]	Netherlands	Interviews and Survey
van Hulst et al. (2020)	Eight scientists and 7 farmers	North East of Scotland.	Semi-structured interviews, co-constructed mental models
Christen et al. (2015)	Nine non-farmer and 8 farmer participants	Rural Scotland	Semi-structured interviews, workshops, on-farm interviews
Okello et al. (2019)	One hundred and two farmers	Six Kenya districts: Buuri, Igembe Central, Igembe South, Laikipia East, Meru Central, and Tigania East	Semi-structured interviews, laddering technique
Krithika et al. (2023)	Fifty participants (farmers, farm laborers and other key stakeholders)	Coimbatore and Tiruvallur districts of Tamil Nadu in India.	Focus group discussions and key informant interviews were done to collect data qualitatively through an open-ended questionnaire which was pre-tested during the pilot survey
Topp et al. (2024)	Five hundred and ninety farmers	Morocco, Spain, and Tunisia	Questionnaire, Semi-structured interviews, survey
D'Agostino et al. (2020)	Twenty two participants (policy makers, water regulators, farmers and representatives from the agrifood industry, environmentalists, academics and researchers)	Malta, Europe	Three-staged approach: Delphi analysis, fuzzy cognitive mapping, questionnaires, backcasting workshop
Mikołajczak et al. (2022)	Thirty six farmers and farming representative	England	Semi-structured interviews
Goswami et al. (2024)	Thirty purposively selected households	Satjelia Island, Coastal region of the Indian Sundarbans	Personal structured interview
Busse et al. (2021)	Twenty three informants (banking sector, real estate agencies, consultancies, law firms and interest organizations, possessing expert knowledge in the fields of Swedish agriculture and forestry)	Northern Germany (Federal State of Lower Saxony and Federal State of Brandenburg)	Semi-structured interviews
Allen et al. ((2018)	Forty stakeholders	Anacostia, Columbia, Middle Rio Grande, and Platte River Basins, all located within the United States	Survey
Adaawen (2021)	Fifty seven in-depth interviews (40 males, 17 females) and surveys with 120 households (81 males, 39 females)	Bongo District, Upper East Region (UER) of North-eastern Ghana	Focus group interviews
Alomia-Hinojosa et al. (2023)	100 households in Palpa (*n =* 50) and Dadeldhura (*n =* 50)	Mid-hill regions of Nepal: Palpa district is located in the Western region and Dadeldhura district located Far-Western region.	Participatory Farm System Mapping and Semi-Structured Interviews
Han et al. (2022)	Eight hundred and eighty one farmers	Michigan and Ohio, USA	Survey
Nyadzi et al. (2021)	Twelve farmers	Kumbungu District. Northern Ghana	Participatory approach using mental models, semi-structured interviews, and workshops
Findlater et al. (2018)	Ninety commercials grain farmers	South Africa's Western Cape	Interview
MacLeod et al. (2022)	One hundred and thirty three respondents	Manaaki Whenua—Landcare Research, New Zealand's national environmental research institute, New Zealand	Mixed-methods research (online surveys, workshops, seminars, meetings, and emails)
Douglas et al. (2016)	Ten irrigators participated in the MIA workshop and 13 in the CIT workshop.	Riverina (Murrumbidgee Irrigation Area—MIA), New South Wales and Riverland, South Australia	Workshop interview
Clements et al. (2021)	Thirty three farmers and 16 outreach professionals	Northern New England	Mental Modeling interviews
Yageta et al. (2022)	Fifty nine farmers	Kitui County, Kenya	Interview
Friedrichsen et al. (2020)	Forty three (farmers, maintainers, local politicians, Constructed wetland neighbors, extension agents, and scientists)	South India	Semi-structured interviews
Hoffman et al. (2014)	Eight hundred and eighty two respondents	Central Coast, Lodi, and Napa Valley	Mail survey
Steger et al. (2021)	Forty one (Guassa Committee (*n =* 27, three each from nine communities), scientists (*n =* 6), local administration officers (*n =* 5), and the Guassa Conservation office (*n =* 3).	Guassa Community Conservation Area, Amhara Region of Ethiopia	Iterative process of constructing and revising mental models through workshops

**Source**: Author's compilation (2024).

Various software tools have been used to understand and represent complex mental models and decision-making processes (Table 5). Among these, Mental Modeler is the most widely used software for mapping and analyzing the differences in individual mental models (Arroyo-Lambaer et al., 2022). It was applied in combination with Fuzzy Logic Cognitive Mapping (Krithika et al., 2023). It has also been applied in the translation of qualitative models to semi-quantitative dynamic models of agricultural systems, as well as in calculating the descriptive statistics of mental models (Clements et al., 2021). This has enabled the construction of group mental models using participatory modeling methods (Douglas et al., 2016; Steger et al., 2021). There is a lack of a dynamic model that can handle feedback loops, uncertainty and real time data updation providing a future scope.

**Table 5:** Software used for modeling cognitive maps in agriculture.

**Software**	**No. of articles**	**References**
Mental Modeler	6	Arroyo-Lambaer et al., 2022; Douglas et al., 2016; Goswami et al., 2020; Krithika et al., 2023; Nyadzi et al., 2021; Steger et al., 2021
FCMapper	4	Christen et al., 2015; Khumairoh et al., 2024; Mehryar et al., 2017; Müller et al., 2019
Cmap software	2	Cordeiro et al., 2021; Salliou and Barnaud, 2017
Micmac software	2	(Ben Mohamed et al., 2024; Mohamed et al., 2024)
Gephi	2	Soto et al., 2021; Topp et al., 2024
FuzzyDances	1	Aravindakshan et al., 2021
UCINET	1	Goswami et al., 2024
Vensim	1	Sanga and Koli, 2023
Decision Explorer	1	Van Winsen et al., 2013
Microsoft Visio©	1	Goller et al., 2021
KH Coder	1	Yageta et al., 2022

**Source**: Author's compilation (2024).

Other Software utilized include FCMapper, which was used to simulate the impact of policy options on the agricultural system (Mehryar et al., 2017; Müller et al., 2019). It has also been used to combine cognitive maps together from focus group discussions (Christen et al., 2015). The conceptual models that portray the knowledge base of the farm system were designed using the Cmap software, which was developed by the Institute for Human and Machine Cognition (IHMC) (Salliou and Barnaud, 2017; Cordeiro et al., 2021). The mind maps were analyzed via the Micmac software. This software was used to conclude the centrality analysis of the variables that affecting the pesticide use (Ben Mohamed et al., 2024). It has also been applied to obtain an understanding of farmers' perceptions and establish cognitive maps of determinants of pro-environmental behavior (Mohamed et al., 2024). The graphical displays of information flows and network visualizations were conducted using the Gephi software (Soto et al., 2021; Topp et al., 2024). Various fuzzy cognitive maps have been created using the FuzzyDances software with specific information on the farmers (Aravindakshan et al., 2021). UCINET 6 network analysis software was utilized for generating level and non-level properties of resource-cycling network (Goswami et al., 2024). The mental model development involved modeling the causal loop diagrams using the Vensim software (Sanga and Koli, 2023). The Decision explorer software was used to analyze the cognitive maps (Van Winsen et al., 2013). The Microsoft Visio software was used to construct and share the mental models during the analysis process (van Hulst et al., 2020). KH Coder software was used to discover the relationship of the soil-knowledge concepts, and visualize farmers‘ perceptions of soil fertility (Yageta et al., 2022).

## 4 Critical gaps and future research directions

### 4.1 Methodological gaps

The specific regional focus and small sample sizes has been mentioned as constraints in many studies. Small or non-random samples can lead to skewness in results, reduce representativeness, and refine communication strategies. This emphasizes the need for a sampling strategy to capture diverse farmer perspectives and ensure reliable inferences. Difficulties in integrating multidisciplinary perspectives, capturing dynamic and non-linear relationships in environmental systems are constraints. These hindrances have to be rectified to gain better understanding of the complex interdependencies with socio-ecological systems in which human behavior, climate change and policy decisions interact. Since there is no standardized procedure that currently exists, future researchers shall develop standard methods for constructing cognitive maps (Van Winsen et al., 2013). A uniform methodology would improve comparison between studies and reduce the researcher bias in mapping mental models. Most cognitive studies, capture behavior at a single time period and thus ignore temporal variability in cognition. Therefore, the integration of longitudinal or seasonal follow-ups would offer a better understanding of dynamic cognitive patterns. Also, the effective use of participatory monitoring and evaluation process in social learning remains underexplored (Soto et al., 2021). This encourages stakeholder involvement, and validate its potential for adaptive resource management. Collective risk perception can also be derived through cognitive mapping (Mikołajczak et al., 2022). Additionally, there is an emphasis on agent-based modeling, which will help improve familiarity and the capability to deal with complex agricultural systems (Mehryar et al., 2019).

### 4.2 Theoretical gaps

The long-term effect of anxiety on the investment decisions of agricultural technology is limited. This shows the importance of cognitive mapping in understanding farmers' decision-making influenced by emotional and survival factors (Findlater et al., 2018). Integrating emotional dimensions can add new dimensions to the theoretical models of farmer behavior under uncertainty. There is a lack of cognitive mapping studies with a cross-cultural focus, longitudinal studies, and gendered mental models. These critical dimensions are important for understanding the socio-cultural variation in perceptions and adaptive behaviors. It may also be used to explain changes in belief patterns. The combination of individual and group level modeling processes is likely to provide a more robust model of the dynamics of socio-cultural processes (Steger et al., 2021). The link between mental models and farmers' behaviors could be ascertained through longitudinal studies with a view to elicit inconsistencies in the mental models (Binder and Schöll, 2010).

### 4.3 Contextual gaps

**Figure 10:**
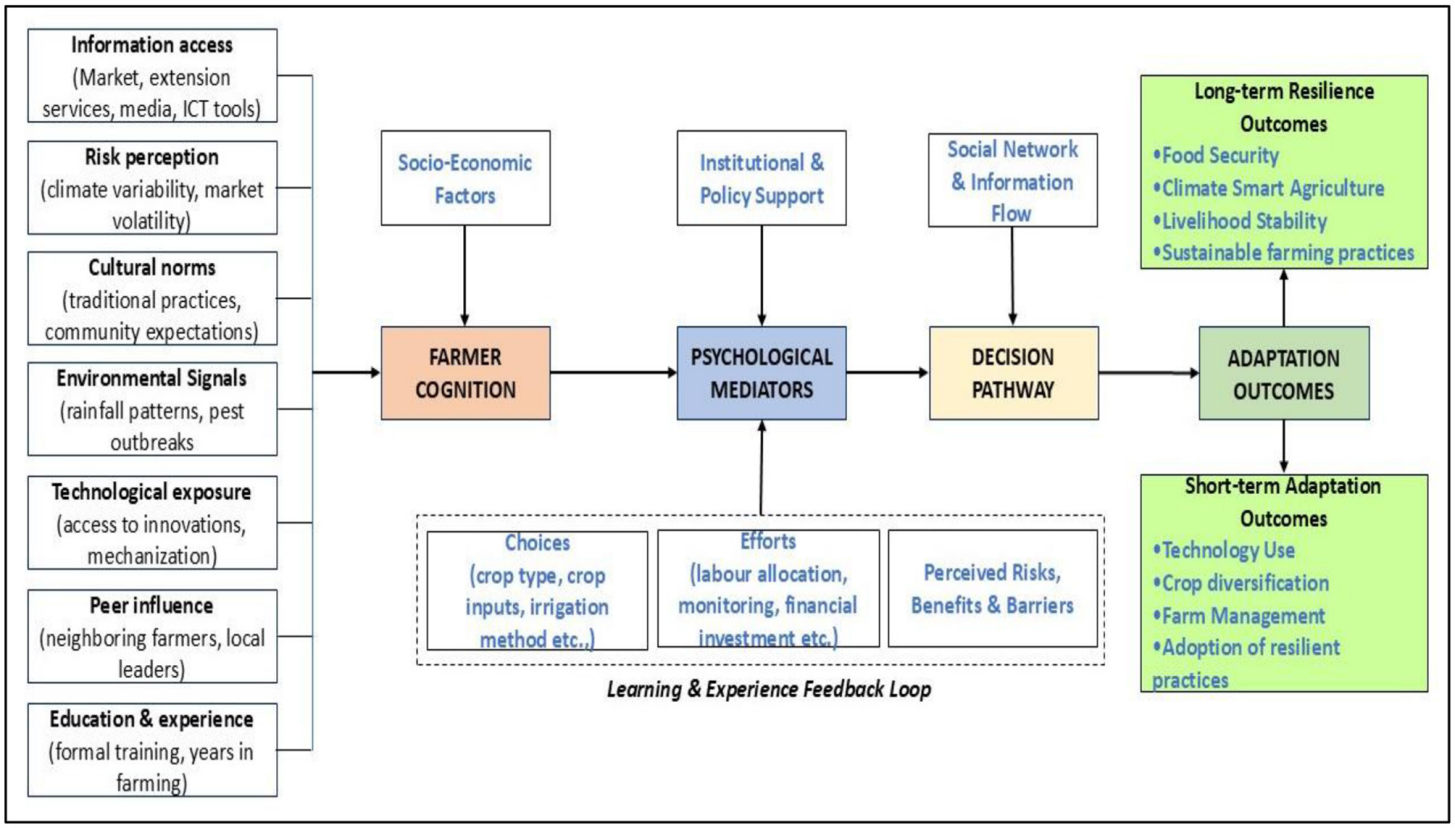
Conceptual map of agricultural decision making. **Source**: Author's compilation (2025).

There is a lack of evidence-informed actions for conservation management which suggest that behavioral insights should be incorporated into conservation policy (MacLeod et al., 2022). This incorporation is critical because policy effectiveness depends on more than economic incentives but also the congruence of socio-cultural derived farmer's beliefs, motivations and risk perceptions. It is important to comprehend uncertainties linked with government policies. This highlights a gap for utilizing mental models to address the perceived constraints of all stakeholders (Douglas et al., 2016). Farmers' mental models can be combined with scientific knowledge to improve soil management (Yageta et al., 2022). This may help to stimulate local experiential knowledge aimed at identifying context specific sustainable practices. Moreover, there have been studies involving in strategies, behavior, and perceptions of community members for the maintenance of wetlands to facilitate ecological functioning. This have demanded the redesigning of constructed wetlands to facilitate intuitive maintenance of communities to enhance their long-term effectiveness and to overcome socio-cultural barriers (Friedrichsen et al., 2020, 2021). The farmers' social representations should be integrated into agricultural policy design to improve landscape stewardship and benefit both ecosystems and society (Vuillot et al., 2020). However, the inter-relationship between farmers' mental models and their practices are seldom considered and the inter-individual differences in management practices can be explored (Vuillot et al., 2016). Knowledge gaps of farmers are a major hindrance in implementing complex agricultural systems, and therefore policies should focus on developing effective intervention strategies (Khumairoh et al., 2024). There is also a need for further empirical research on the effects of digitalization on the experience and knowledge of farmers has possible scope (Goller et al., 2021). The impact of technological innovations on labor supply and productivity in the agricultural food supply chain can also be studied (Oliveira-Dias et al., 2022). Furthermore, the focus on broadening stakeholder networks and the implementation of cultural, social, and psychological perspectives, including risk management strategies, training, and policy forms will be beneficial in promoting sustainable resource management (Figure 10). Cognitive mapping outputs can significantly enhance climate advisory systems by translating farmers' perceptions, experiential expertise and adaptive practices into digital decision support systems (DSS) and weather-based advisories. These maps can provide granular insights on region specific cropping decisions that can improve the precision of localized advisory logic. When these DSS recommendations reflect farmers' cognitive frames, their relevance and adoption will increase. This can improve participatory updates and mobile feedback loops that enables dynamic system learning. Also, this integration supports co-evolution between farmer decision-making and advisory algorithms, resulting in a context-specific climate resilience. By addressing the future trends, the agricultural sector can be driven toward enhanced resilience, efficiency, and sustainability.

## 5 Conclusion

The research problem that was addressed in this review is of high importance, as it concerns cognition of farmers that have been proven useful in decision-making processes within farming systems. The mental models, perception of risks, and cognitive biases were found to significantly influence agricultural decisions. Cognitive biases like the availability bias, optimism bias, confirmation bias, status quo bias and social norms bias may cause the individuals to rely on what has happened recently, to underestimate risk, stick to the norm and to trust peers more than facts. The biases may be mitigated with the aid of the extension methods that offer the relevant local data, allow low-risk trial, simplify the decision-making, rely on peer learning and trusted people, and resort to behavioral nudges. This could highlight short term gains whilst projecting targeted behaviors. The idea is not to alter communication and engagement mechanisms, but to organize these activities in the light of the knowledge about the manner, in which people think, and what they do to enhance the adoption of the sustainable agricultural practices. Moreover, to build climate resilient agriculture, there is a need to change individual farmer mentality to appreciate resilience to be a social, collective process. Social networks provide community learning systems that support quick and fair transfer of adaptive information, and enhance communal capacity to act to climate shocks. Agricultural communities are in a position to not only come out of a crisis, but be able to adapt and change in the face of adversity with time. There is also the need to know that the cognitive processes of a farmer may be used in the designing of extension modules which may reveal the way farmers perceive an information. This allows broadening of opportunities, and advice on individual decision-making. This also makes it easier to incorporate cognitive processes in climate adaptation policies in order that the interventions may accommodate differences in real-world behavior in practices of adoption. These findings give a significance to the use of cognitive mapping tools that are sensitive to the numerous complex factors influencing the decisions of farmers. It can also be useful in coming up with effective and sustainable agricultural practices. Thus, Cognitive mapping allows us to understand how farmers think and influence resilient agriculture. These insights can help policymakers integrate the cognitive realities of the farmers in a manner that is inclusive and effective, therefore, ensuring sustainability.

## Data Availability

The original contributions presented in the study are included in the article/supplementary material, further inquiries can be directed to the corresponding author.
